# New insights into Sirt1: potential therapeutic targets for the treatment of cerebral ischemic stroke

**DOI:** 10.3389/fncel.2023.1228761

**Published:** 2023-08-09

**Authors:** Hao Tang, Jun Wen, Ting Qin, Yue Chen, Jiagui Huang, Qinghuan Yang, Peiran Jiang, Ling Wang, Yong Zhao, Qin Yang

**Affiliations:** ^1^Department of Neurology, The First Affiliated Hospital of Chongqing Medical University, Chongqing, China; ^2^Department of Respiratory Disease, Daping Hospital, Army Medical University, Chongqing, China

**Keywords:** Sirt1, cerebral ischemic stroke, neuroprotection, deacetylation, programmed cell death

## Abstract

Ischemic stroke is one of the main causes of mortality and disability worldwide. However, the majority of patients are currently unable to benefit from intravenous thrombolysis or intravascular mechanical thrombectomy due to the limited treatment windows and serious complications. Silent mating type information regulation 2 homolog 1 (Sirt1), a nicotine adenine dinucleotide-dependent enzyme, has emerged as a potential therapeutic target for ischemic stroke due to its ability to maintain brain homeostasis and possess neuroprotective properties in a variety of pathological conditions for the central nervous system. Animal and clinical studies have shown that activation of Sirt1 can lessen neurological deficits and reduce the infarcted volume, offering promise for the treatment of ischemic stroke. In this review, we summarized the direct evidence and related mechanisms of Sirt1 providing neuroprotection against cerebral ischemic stroke. Firstly, we introduced the protein structure, catalytic mechanism and specific location of Sirt1 in the central nervous system. Secondly, we list the activators and inhibitors of Sirt1, which are primarily divided into three categories: natural, synthetic and physiological. Finally, we reviewed the neuroprotective effects of Sirt1 in ischemic stroke and discussed the specific mechanisms, including reducing neurological deficits by inhibiting various programmed cell death such as pyroptosis, necroptosis, ferroptosis, and cuproptosis in the acute phase, as well as enhancing neurological repair by promoting angiogenesis and neurogenesis in the later stage. Our review aims to contribute to a deeper understanding of the critical role of Sirt1 in cerebral ischemic stroke and to offer novel therapeutic strategies for this condition.

## Introduction

Currently, ischemic stroke is one of the well-known leading causes of mortality and long-term disability globally (Ahmadi et al., 2020). Although substantial efforts have been made to search for better treatment modalities for ischemic stroke, remarkably few strategies are considered sufficiently effective due to their complex pathophysiological mechanism, including excitotoxicity, oxidative stress, inflammation and blood-brain barrier (BBB) damage.

At present, thrombolysis and mechanical thrombectomy are the only authorized treatments for acute ischemic stroke clinically. However, its therapeutic application is severely constrained by the narrow time windows and secondary injury caused by vascular recanalization (Nogueira et al., 2018; Turc et al., 2019). Therefore, it is essential to seek for new alternatives that can prolong the time windows and improve the prognosis of ischemic stroke patients. Among the potential therapeutic targets, Silent mating type information regulation 2 homolog 1 (Sirt1) merits special attention. Because it can not only lessen the neurological injury in the acute phase, but also enhance neurorestoration in the later stage.

Sirt1, a nicotine adenine dinucleotide (NAD^+^)-dependent enzyme, is the member of the sirtuins family, which can catalyze the deacetylation of histone and non-histone substrates (such as P53, FOXO3), and plays a crucial role in chromatin remodeling, gene regulation and metabolism (Meng et al., 2020). Sirt1 is abundant in early embryo and widely expressed in mature tissues (Chang and Guarente, 2014). In the central nervous system (CNS), Sirt1 is extensively expressed in neurons, neural stem cells, neural precursor cells, astrocytes and microglia of embryonic and adult brains. Further studies shows that Sirt1 is involved in the modulation of neurodevelopment, learning, memory and metabolic function (Chang and Guarente, 2014; Herskovits and Guarente, 2014; Zhou et al., 2018). It has been discovered that activated Sirt1 exhibits obviously potent neuroprotective effects on ischemic stroke and other neurodegenerative diseases.

In the review, we summarized the protective effects of Sirt1 on ischemic stroke and its related mechanisms, including reducing inflammatory response, inhibiting oxidative stress and ultimately modulating programmed cell death in the acute phase, and promoting neurological functional recovery through enhancing angiogenesis and neurogenesis in the later stage. The review may provide a fundamental basis for the design of new drugs for ischemic stroke.

## Sirt1 protein structure

**FIGURE 1:**
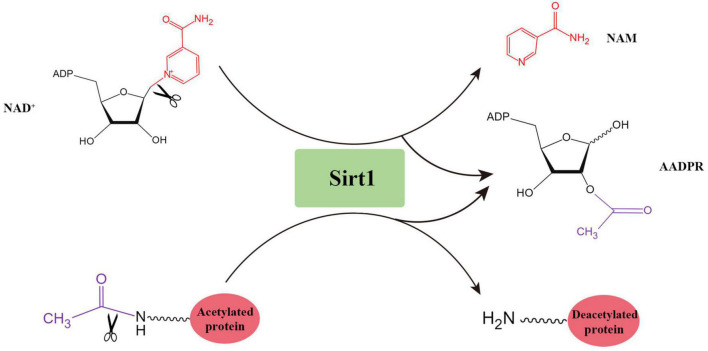
The deacetylation reaction catalyzed by Sirt1.

Sirtuins are a group of highly conserved NAD^+^-dependent deacetylases. Mammalian sirtuins can be split into seven members (Sirt1∼7) according to their structure and function. Sirt1 is firstly discovered and the most studied. The human Sirt1 protein (747 amino acids) is composed of highly conserved catalytic domain, N-terminal domain and C-terminal domain. For human Sirt1, the catalytic core consists of two domains. The larger NAD^+^-binding domain consists of a Rossmann fold, and the smaller domain composes of a helical structure and a zinc-binding module. The Sirt1-mediated catalytic reaction is initiated by the binding of acetylated residues of the target molecule with NAD^+^ through the cleft between these two domains (Sauve et al., 2006), which eventually produces the deacetylated substrates, nicotinamide and 2′-O-acetyl-ADP-ribose (AADPR) (Tanner et al., 2000; Figure 1).

## Sirt1 protein localization

**FIGURE 2:**
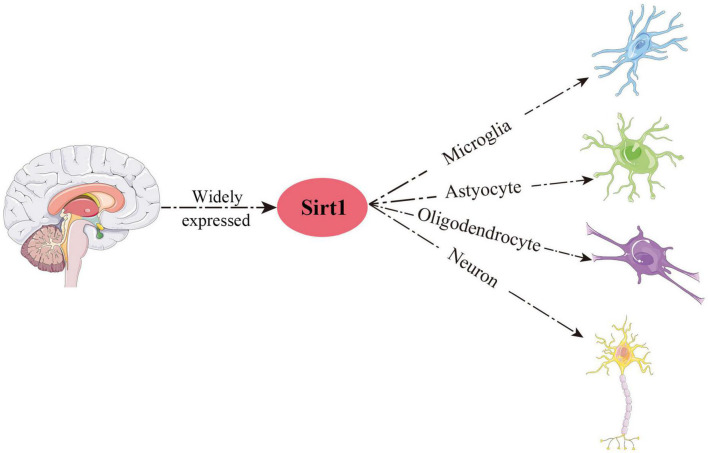
Schematic overview of the main distribution of Sirt1 in the CNS.

Growing studies have verified that Sirt1 is widely distributed in human and rodent organs, including brain, heart and liver (Sakamoto et al., 2004; Tanno et al., 2010; Ogawa et al., 2011; Al-Bahrani et al., 2015; Cao et al., 2018). An anatomical study of rodent and human nervous system showed that Sirt1 was localized in the regions of the hippocampus, prefrontal cortex and basal ganglia (Zakhary et al., 2010). Subsequently, Sirt1 was also found to express in hypothalamus and cerebellum (Ramadori et al., 2008). In addition to neurons, Sirt1 has also been demonstrated to be expressed in various glial cells (Kannan et al., 2013; Prozorovski et al., 2019), such as microglia, astrocytes and oligodendrocytes (Figure 2). In summary, Sirt1 is widely distributed in the CNS.

Next, let us turn to the subcellular localization of Sirt1. The nuclear localization signal is found on residues 41–46 of the Sirt1 protein (Frye, 1999). Therefore, it makes sense that Sirt1 is classified as a nuclear protein. However, a variety of findings suggested that Sirt1 was also present in cytoplasm under certain conditions (Jin et al., 2007; Hisahara et al., 2008; Yu et al., 2020). Subsequent study confirmed that Sirt1 could shuttle between the nucleus and cytoplasm, which was mediated by the nuclear import and export sequences in the N-terminal region of Sirt1 (Tanno et al., 2007).

## Activators and inhibitors of Sirt1

Sirt1 plays an important role in various physiological and pathological processes in organisms, and up or down regulation of Sirt1 often means completely different outcomes. Therefore, searching for the activator and inhibitors of Sirt1 remains significantly important for the prevention and treatment of various diseases, including cerebral ischemic stroke. Next, we will simply divide them into three categories: natural, synthetic and physiological (Table 1).

**TABLE 1:** The major activators and inhibitors of Sirt1.

Sirt1 activators/inhibitors	Categories	Catalytic mechanism	References
**Activators**			
AROS	Physiological	Binding to the Sirt1 catalytic domain	Autiero et al., 2008
LARP7	Physiological	Strongly binding to the N-terminal domain of Sirt1	Yan et al., 2021; Yu H. et al., 2021
Resveratrol	Natural	(1) Binding with the N-terminal domain of Sirt1 and the related substrate at the same time (2) Interacting with LAMIN A	Liu et al., 2012; Cao et al., 2015
Quercetin	Natural	Binding to the helix2-turn-helix3 motif in the N-terminal domain of Sirt1	Zhang et al., 2021
Curcumin/Salvianolic acid B	Natural	Uncertain. Further studies are need to clarify the specific catalytic mechanism	Lv et al., 2015; Zhang et al., 2017; Ling et al., 2018
SRT1720	Synthetic	Binding to the Sirt1-substrate complex at the allosteric site of the amino terminal catalytic region	Milne et al., 2007
**Inhibitors**			
DBC1	Physiological	Binding to the catalytic domain of Sirt1	Kim et al., 2008
Suramin	Natural	Tightly binding to Sirt1 catalytic domain, and simultaneously occupy the binding region of NAD + and the substrate	Trapp et al., 2007
EX527	Synthetic	Specifically binding to Sirt1 to inhibit the formation of the Sirt1-substrate complex	Gertz et al., 2013

### Physiological

Active regulator of Sirt1 (AROS) is seen as a direct interactant of Sirt1, which functions by directly binding at a site (amino acids 114–217) distal to the Sirt1 catalytic domain, thereby promoting Sirt1 deacetylation activity (Autiero et al., 2008). Kim et al. (2007) found that AROS can upregulate Sirt1 activity so as to inhibit P53-dependent transcriptional activation by directly binding to Sirt1. Nevertheless, AROS is considered as a weak activator of Sirt1, which requires strict conditions for this activation. The regulation of P53 acetylation by AROS depends to some extent on the cell context. Specifically, AROS suppressed the acetylation of P53 only during the process of cell damaging stress (Knight et al., 2013). Different from AROS, member of La ribonucleoprotein domain family 7 (LARP7) is an RNA binding protein and a strong activator of Sirt1, which has been reported to play a positive role in aging and heart failure by regulating Sirt1 (Yan et al., 2021; Yu H. et al., 2021). LARP7 strongly bound to Sirt1 residues 158–225 and allosterically enhances Sirt1 deacetylase activity, thereby inhibiting the acetylation of P53 and P65, respectively.

Conversely, Deleted in Breast Cancer-1 (DBC1) was reported to be a physiological inhibitor of Sirt1, which could directly interact with Sirt1 and suppress its activity (Kim et al., 2008). Deletion analysis revealed that the inhibition of Sirt1 deacetylation activity was attributed to DBC1’s direct binding to the catalytic domain of Sirt1, which hindered the binding of Sirt1 with downstream molecules P53 and FOXO. And hyperacetylation of P53 and FOXO could augment cellular apoptosis under damaging stress, which may be the ability of DBC1 as a tumor suppressor (Kim et al., 2008).

### Natural

Resveratrol, a natural polyphenolic compound, presents in many plants and is identified as the natural activator of Sirt1, which plays an important role in several CNS disorders. Our previous studies found that resveratrol alleviated cerebral ischemic stroke injury by inhibiting neuronal apoptosis (Yu et al., 2017), attenuating oxidative stress (Shen et al., 2016), promoting synaptogenesis (Yu P. et al., 2021), and suppressing ferroptosis (Zhu et al., 2022). Some researchers also figured out that resveratrol could modulate autophagy (He et al., 2017) and inhibit activation of inflammasomes (Chiang et al., 2022) to improve the ischemic injury. As the activator of Sirt1, resveratrol functions by binding with the N-terminal domain of Sirt1 and the related substrate at the same time, thereby promoting the tighter combination of Sirt1 and substrate and Sirt1 deacetylation activity (Cao et al., 2015). In addition to directly activating Sirt1, resveratrol can also increase the activity of Sirt1 by interacting with LAMIN A, which is a key protein for maintaining nuclear structure (Liu et al., 2012).

Quercetin is a natural flavonoid, which exists in many fruits and vegetables (Cui et al., 2022). A recent study indicated that quercetin maintained the BBB integrity and inhibiting reactive oxygen species (ROS) generation through activating Sirt1, thereby improving neurological function after ischemic stroke (Yang R. et al., 2022). Recently, Zhang et al. (2021) found that quercetin activates Sirt1 activity by binding to the helix2-turn-helix3 motif in the N-terminal domain of Sirt1. Compared with resveratrol, quercetin can more effectively activate Sirt1 deacetylase activity and enhance the binding of Sirt1 to the substrate acetylated P53 (Zhang et al., 2021). Curcumin and salvianolic acid B, also as natural polyphenols, performs the similar characteristics of activating Sirt1 as resveratrol does. The neuroprotective effects mediated by them were mainly attributed by reducing the release of inflammatory factors and cellular apoptosis through activating Sirt1 (Lv et al., 2015; Zhang et al., 2017; Ling et al., 2018). However, although enough studies had confirmed that both of them could activate Sirt1 to exert their neuroprotection, the molecular mechanism of their activation of Sirt1 remains to be clarified.

Conversely, Suramin, extracted from pine needles, was first used to manage trypanosomiasis and nematode disease, and was later reported to have certain anti-tumor activity and anti-apoptosis effects. The co-crystal structure analysis showed that suramin can tightly bind to Sirt1 catalytic domain, and simultaneously occupy the binding region of NAD + and the substrate, thereby inhibiting Sirt1 deacetylase activity (Trapp et al., 2007).

### Synthetic

High-throughput screening found that SRT1720 was a potential Sirt1 activator, and it performed a much stronger property to activate Sirt1 than resveratrol. Similar to resveratrol, SRT1720 binds to the Sirt1-substrate complex at the allosteric site of the amino terminal catalytic region, thereby promoting Sirt1 deacetylation activity (Milne et al., 2007). It was reported that application of SRT1720 could provide the neuroprotective effects by regulating autophagy (Bai et al., 2021), inhibiting neuroinflammation (Wang F. et al., 2019) and promoting microglia polarization (Xia et al., 2021) through activating Sirt1. Similar to SRT1720, SRT2104 was also found to efficiently activate Sirt1 to alleviate brain damage after ischemic stroke by regulating microglia polarization (Fu et al., 2021).

Conversely, several chemical compounds have performed their ability to suppress Sirt1 activity. Since its discovery in 2005, EX527 has become one of the most effective selective inhibitors of Sirt1. EX527 can specifically bind to Sirt1 to inhibit the formation of the Sirt1-substrate complex, which leads to the acceleration of substrate acetylation (Gertz et al., 2013). In addition to EX527, sirtinol is also the effective inhibitors of Sirt1 and involved in the development of cerebral ischemic injury and neurological damage (Tang et al., 2017).

## Crucial role of Sirt1 in neurodegenerative diseases

Neurodegenerative diseases, such as Alzheimer’s disease (AD), Parkinson’s disease (PD), and Huntington’s disease, are chronic conditions characterized by neuronal dysfunction and loss. Recent studies have highlighted the role of Sirt1 in regulating synaptic plasticity and mitigating neurodegenerative damage within the CNS. The levels of Sirt1 protein were found to be significantly reduced in patients with neurodegenerative diseases compared to those undergoing normal aging, suggesting that diminished Sirt1 expression and activity contribute to the pathological progression of these conditions (Cao et al., 2018). Moreover, overexpression of Sirt1 can modulate the impact of Aβ in AD and impede the formation of synuclein aggregates in PD. Conversely, the inactivation of Sirt1 has shown potential to ameliorate the mitochondrial apoptosis pathway, which is implicated in the pathogenesis of aging, metabolic disorders, and neurodegenerative diseases (Rana et al., 2019). In summary, Sirt1 may play a crucial role in neurodegenerative diseases.

## Neuroprotective role of Sirt1 for ischemic stroke

As a survival factor against the aging process, Sirt1 has been shown to exert neuroprotective effects in the neurodegenerative diseases, such as AD, PD, and Huntington’s disease. Recent studies have found that Sirt1 can alleviate ischemic stroke injury, including reducing cerebral infarcted volume and neurological deficits. Next, we will discuss the progresses of Sirt1 in animal models and clinical trials of ischemic stroke in recent years.

### Clinical trials

In 2021, a case-control study showed that the activity of Sirt1 in the serum of patients with acute ischemic stroke (AIS) was significantly lower than that of the control group, and its levels were significantly negatively correlated with the stroke score, which suggested that Sirt1 could be used as a potential biomarker for predicting the risk of AIS (Esmayel et al., 2021). However, another clinical trial reached the opposite conclusion. The researchers found that Sirt1 activity increased sharply after ischemic stroke, and there was no significant correlation between its activity and stroke score, which blocked its opportunity as a biomarker for prognosticating the functional outcome of AIS patients (Liu et al., 2018). These studies suggest that Sirt1 expression may be a dynamic process after stroke, so further study with larger sample and more accurate grouping was needed to clarify its role in ischemic stroke.

Subsequently, a cohort study focused on evaluating the effects of Sirt1 activator resveratrol on blood pressure, weight status, glucose, and lipid profile which are the main risk factors for ischemic stroke. It was found that resveratrol can significantly reduce these parameters at 6 and 12 months after the initial evaluation, which suggested that resveratrol could serve as the promising drug to prevent ischemic stroke (Fodor et al., 2018). Another clinical trial also reported the neuroprotective effects of resveratrol on AIS patients. As the most effective method to treat ischemic stroke, recombinant tissue plasminogen activator (r-tPA) is severely limited by its narrow therapeutic window. Chen et al. (2016) found that resveratrol can prolong the clinical therapeutic window of r-tPA and reduce the MMP-induced neurological deficits, thus improving the prognosis of AIS patients receiving r-tPA treatment at a later stage.

These clinical evidences suggest that Sirt1 has potential as a target for prevention and treatment for AIS patients, and can be used as a prognostic indicator of ischemic stroke.

### Animal studies

Similar to the results of clinical trials, Sirt1 expression in rodent models was modulated by ischemic injury as well. For instance, Sirt1 was upregulated significantly in ischemic penumbra from 18 h to 7 days after ischemic stroke (Hernández-Jiménez et al., 2013). However, another study reached the opposite conclusion. They found that compared with control group, the level of Sirt1 in middle cerebral artery occlusion (MCAO) group decreased sharply (Kalaivani et al., 2014). The huge difference in results may be attributed to different species and different model construction used in such two studies.

In order to further clarify the role of Sirt1 in cerebral ischemic/reperfusion (I/R) injury, researchers constructed its overexpression and knockout model through genetic manipulation technology. Compared with wild-type model, Sirt1^–/–^ mice subjected to permanent MCAO performed the larger infarct size (Hernández-Jiménez et al., 2013). Conversely, overexpressing Sirt1 could reduce hippocampal injury after bilateral common carotid artery occlusion (Hattori et al., 2015). In summary, activation of Sirt1 has neuroprotective effects and regulates the outcome of cerebral ischemic injury.

## Potential mechanisms of Sirt1 for ischemic stroke

**FIGURE 3:**
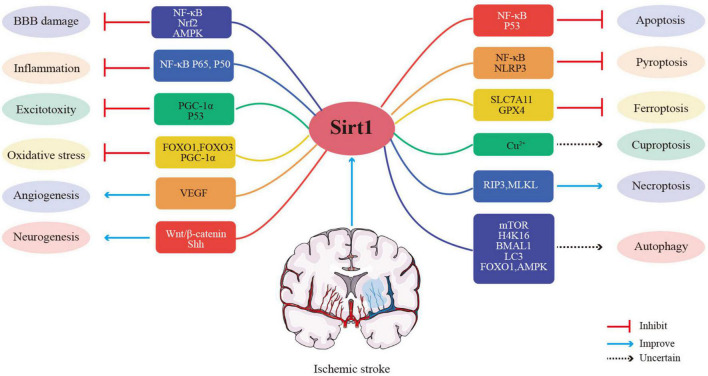
Potential mechanisms of Sirt1 for ischemic stroke.

Studies in animal models and clinical trials have shown that Sirt1 is an efficient treatment for ischemic stroke. How does Sirt1 play a therapeutic role? What are the therapeutic targets for Sirt1? The specific mechanisms of Sirt1 for regulating cerebral ischemic stroke will be discussed in the following section (Figure 3).

### Anti-oxidative stress

Oxidative stress is one of the earliest outcomes in the period of ischemic stroke, causing cascades of cellular and molecular processes that leads to neurodegeneration and death of neurons. Increased levels of ROS in cells, such as hydroxyl radicals, can result in oxidative stress and mitochondrial dysfunction, which can lead to cerebral ischemia and further aggravate the cerebral injury. Sirt1 has been identified as playing an essential role in oxidative stress. Sirt1 is activated after the onset of stroke and can regulate multiple signaling pathways to affect oxidative stress, further modulating the pathological process of stroke.

The anti-oxidant properties of Sirt1 rely basically on targeting the FOXO transcription factors. Specifically, FOXO3 has been demonstrated to play an essential role in the regulation of oxidative stress, which can upregulate the expression of several antioxidant proteins, including superoxide dismutase, manganese superoxide dismutase, and catalase. FOXO3 can be phosphorylated and deacetylated to regulate its transcriptional activity.

In addition, deacetylation of FOXO3 by Sirt1 can prevent cell death induced by FOXO3. Zhang et al. (2022) found that Bergenin hampered the production of inflammatory factors and oxidative stress mediators by boosting the Sirt1/FOXO3 pathway. Similarly, the lncRNA SNHG12 also performed the anti-oxidant effects in ischemic model by activating Sirt1/FOXO3 pathway.

In addition, FOXO1 and FOXO3 can regulate the level of peroxisome proliferator-activated receptor gamma co-activator 1-α(PGC-1α). PGC-1α is involved to the oxidative phosphorylation and ROS detoxification, contributing to maintaining metabolic homeostasis. PGC-1α upregulation could reduce the oxidative stress-mediated neuronal death (St-Pierre et al., 2006). Reversely, PGC-1α depletion further increased the cellular injury induced by oxidative stress (Pérez et al., 2019). Moreover, Calycosin-7-glucoside reduced neuronal death mediated by oxidative stress through activating Sirt1/FOXO1/PGC-1α signaling pathway (Yan et al., 2019). Similarly, Xie et al. (2020) found that notoginseng leaf triterpenes, a natural ingredient, suppressed the excessive oxidative stress and mitochondrial damage at least partly via Sirt1/FOXO3/PGC-1α axis. These studies suggest that Sirt1 has anti-oxidant stress effect.

### Anti-inflammation

Inflammatory response plays a crucial role in the pathophysiology of stroke because it runs through the whole process. The nuclear factor kappa B (NF-κB) is a major transcription factor of inflammation, which can be specifically activated after cerebral ischemic stroke. Sirt1 can alleviate cerebral ischemia/reperfusion injury by regulating NF-κB pathway. For example, Sirt1 regulated the transcriptional activity of NF-κB by directly deacetylating NF-κB P65, thereby modulating the expression of inflammatory cytokine TNF-α (Yeung et al., 2004). Sirt1 activator resveratrol could reduce OGD/R-mediated neuronal death and neuroinflammation by regulating NF-κB p50 deacetylation (Lanzillotta et al., 2010). Besides acetylation modification, Sirt1 can also mitigated NF-κB phosphorylation to alleviate microglia inflammation (Hu et al., 2022). In addition, Sirt1 can indirectly modulate NF-κB pathway through other targets, including TLR4, FOXO3, Nrf2 and so on. Specifically, Bergenin inhibited the expression of inflammatory factors in MCAO model via Sirt1/FOXO3/NF-κB pathway (Zhang et al., 2022). TLR4 was also involved in the regulation of Sirt1 on NF-κB-mediated inflammatory response (Le et al., 2019). And Sirt1 can modulate Nrf2-NF-κB signaling pathway thereby reducing inflammatory respose and protect neurons from OGD damage (Zheng et al., 2022). Taken together, Sirt1 can downregulate the inflammatory response after cerebral ischemia by directly or indirectly regulating NF-κB signaling pathway.

### Affecting excitotoxicity

Glutamate is a primary excitatory amino acid neurotransmitter and activation of glutamate receptors including *N*-methyl-D-aspartate (NMDA) receptor plays crucial roles in the central nervous system. However, excessive NMDA receptor can result in intracellular calcium overload, leading to an enzymatic cascade of events resulting ultimately in cell death known as excitotoxicity. NMDA-mediated excitotoxicity has been associated with a variety of nervous system diseases, including stroke and epilepsy. Therefore, better management of excitotoxicity is of great significance for maintaining brain homeostasis and alleviating neurological damage after cerebral ischemic stroke.

The recent study suggested that Ca-PKC-HuR-Sirt1 axis was involved in the glutamate-mediated excitotoxicity, and Sirt1 is the key node (Yang et al., 2020). Subsequently, it was reported that Sirt1 protected cerebral cortical and hippocampal neurons from glutamate-induced injury, which was mainly due to its ability to deacetylate PGC-1α (Jia et al., 2016; Yue et al., 2016). Interestingly, Yang et al. (2017) found that Sirt1 activator resveratrol could also shield cortical neurons from glutamate-induced excitotoxicity through suppressing P53 acetylation. Moreover, the inhibition of Sirt1 on excitotoxicity has also been verified in rodent model. Recent reportedly, α-amino-3-hydroxy-5-methyl-4-isoxazolepropionic acid-mediated excitotoxicity led to a progressive motor neuron degeneration and motor deficits. Quercetin could improve these neurological deficits, and its protective effects was reversed by Sirt1 inhibitor EX527, which implicated the crucial role of Sirt1 on exerting the neuroprotection by suppressing excitotoxicity (Lazo-Gomez and Tapia, 2017).

In summary, these evidences have proved that Sirt1 is a potential target for blocking glutamate-induced excitotoxicity. However, further study is needed to confirm whether and how Sirt1 diminishes excitotoxicity after ischemic stroke.

### Regulating BBB damage

It is well known that BBB is served as the first line of defense to prevent harmful substances from entering the brain and is vital for maintaining brain homeostasis. BBB consists primarily of tightly connected brain microvascular endothelial cells (BMECs), basement membrane, astrocyte end-foot, and pericytes. Disruption of BBB integrity leads to further damage to the brain after ischemic stroke. Increasing evidence suggests that Sirt1 can regulate ischemia-induced BBB damage, thus providing neuroprotection.

BMECs play an essential role in maintaining BBB integrity. CaMKK (α and β), a major kinase activated by elevated intracellular calcium, has been shown to activate Sirt1, a key endothelial protector. Recently, Sun et al. found that CaMKK activation may attenuate ischemic brain injury by protecting the brain microvascular system through Sirt1. Another research implied that lncRNA Snhg8 could relieve ischemic injury of BMECs both *in vitro* and *in vivo* by targeting Sirt1-mediated NF-κB pathway through sponging miR-425-5p. It was suggested that Snhg8/miR-425-5p/Sirt1/NF-κB axis plays a critical role in the regulation of cerebral ischemia-induced BBB damage. Similarly, circHIPK3 acted as an endogenous sponge of miR-148b-3p to decrease its activity, resulting in downregulation of Sirt1 expression and subsequent BMEC apoptosis and mitochondrial dysfunction, further exacerbating BBB damage (Chen G. et al., 2022). In addition, Sirt1 agonist quercetin ameliorates neurological deficits and BBB integrity through Sirt1/Nrf2/HO-1 signaling, and its protective effect is partially reversed by the Sirt1 inhibitor EX527.

Although most investigators have confirmed the positive role of Sirt1 in improving BBB damage in ischemic stroke, there are still some studies that contradict these findings. It was reported that Sirt1 was involved to regulate expression of Sirt3, induction of apoptosis, and production of ROS by inhibiting AMPK-PGC1 pathway, thereby increasing BBB permeability.

Further studies are required to elucidate the specific mechanism of Sirt1 for regulating BBB and thus explain the contradiction in these studies.

### Controlling programmed cell death

Cell death includes uncontrolled accidental cell death and programmed cell death (PCD), which can be activated during trauma, ischemia, hemorrhage, inflammation, oxidative stress, and so on. PCD is induced by one or more signals and can be managed through pharmacological or genetical intervention, including apoptosis, necroptosis, autophagy, pyroptosis and ferroptosis. Recently, cuproptosis has been recognized as a novel PCD. Growing studies have revealed that Sirt1 can alleviate cerebral ischemic injury by regulating the occurrence of PCD. Thus, we provided supporting data that Sirt1 regulated PCD in ischemic stroke.

### Apoptosis

Apoptosis is a programmed cell death process that relies on caspase activity and is characterized by cell shrinkage, membrane blebbing, and chromatin condensation. It can be activated by either the intrinsic or extrinsic pathway (Carneiro and El-Deiry, 2020). Specifically, the pro-apoptotic proteins of the B-cell lymphoma 2 (Bcl-2) family increase the permeability of the outer mitochondrial membrane, which leads to the activation of caspase proteases and eventually, cell disintegration. Sirt1 plays an important role in endogenous neuroprotection against ischemic stroke due to its anti-apoptotic effects (Gao et al., 2022). Inhibition of Sirt1 exacerbates ischemic injury accompanied by increased acetylation of P53 and NF-κB P65, which are important factors in apoptotic pathways that cause brain damage (Hernández-Jiménez et al., 2013).

Maresin 1 (MaR1), a mediator released by M2 macrophages, has been shown to possess anti-inflammatory and anti-apoptotic properties in several diseases (Li et al., 2021; Li H. et al., 2022; Yang W. et al., 2022). In ischemic stroke, MaR1 inhibited apoptosis and reduced injury by up-regulating expression of Sirt1 and Bcl-2 and down-regulating expression of acetylated NF-κB and Bax, Sirt1 inhibitor EX527 could partially reverse the effects, which suggested that the Sirt1/P65 signaling was specifically involved in MaR1-mediated protection against ischemic stroke (Xian et al., 2019). In OGD model of PC12 cells, kaempferol, a natural flavonol, reduces P66shc expression, promotes the deacetylation of P66shc by up-regulating Sirt1, and inhibits cellular apoptosis and mitochondrial dysfunction. This suggested that kaempferol inhibited OGD-mediated apoptosis via Sirt1/p66shc axis (Zhou and Li, 2020).

QIK 6 is a member of the STAR family and has recently been found to be predominantly expressed in primary neurons. Its neuroprotective effects against ischemic stroke have been demonstrated, and Sirt1 is considered the most critical node in this process. On the one hand, Sirt1 induced the deacetylation of QIK 6; on the other hand, Sirt1 activated PPARγ/PGC-1α Signal pathway, both of which could promote synthesis of triglyceride and inhibit neuronal apoptosis, thus slowing the progression of stroke. In a word, Sirt1 mediated the synthesis of triglyceride and inhibition of neuronal apoptosis after stroke, which was associated with the QKI 6 and the PPARγ/PGC-1α signaling pathway (Liu R. et al., 2021).

MicroRNAs (miRs) regulate gene expression by inhibiting protein translation and targeting mRNA destabilization/degradation (Szabo and Bala, 2013). Increasing evidence has indicated that miRs play a key role in various pathological processes, including inflammation, neurodegeneration and cellular apoptosis (Di Leva et al., 2014). It was reported that the miR-149-5p levels were markedly decreased at 24 h after cerebral I/R injury, and Sirt1 natural activator resveratrol could increase its activity accompanied by the downregulation of P53 and caspase-3. This implied that miR-149-5p was involved in the regulation of caspase-3- mediated apoptotic neuronal cell death via Sirt1/P53 axis (Teertam et al., 2020). Another study demonstrated that miR-489-3p was also involved in the regulation of Sirt1 on apoptosis in ischemic stroke (Song et al., 2022). After ischemic stroke, miR-489-3p was upregulated, while Sirt1 was downregulated. Silencing miR-489-3p inhibited neuronal apoptosis and improved neurological function by targeting Sirt1. Moreover, a recent report identified Sirt1 as a target gene of miR-142-3p. The study revealed that miR-142-3p can modulate neuronal apoptosis after ischemic stroke by targeting Sirt1 (Meng et al., 2023). These studies showed that the anti-apoptotic effects of various substances in ischemic stroke are achieved by targeting Sirt1. Therefore, Sirt1 may be one of the key targets for regulating apoptosis.

### Pyroptosis

Pyroptosis is a form of regulated necrosis, triggered by inflammatory Caspase-1 after its activation by various inflammasomes, which can mediate the effect of Gasdermin-D protein, leading to cell lysis and extracellular release of the cytosolic contents and secretion of pro-inflammatory mediators, such as interleukin (IL)-1β and IL-18, resulting in the excessive inflammatory response (Sharma and Kanneganti, 2021). Specifically, The NLRP3 inflammasome is among the most prominent inflammasomes, with high expression levels in the brain, as it plays a crucial role in detecting cell damage and initiating an inflammatory cascade. Several studies have indicated that the NLRP3 inflammasome plays an essential part in the occurrence and development of cerebral I/R injury (Heinisch et al., 2022; Kerr et al., 2022), and the activation of Sirt1 can exert the neuroprotection via inhibition of this pathway (Zhou et al., 2023).

Growing evidence indicates that mesenchymal stem cells (MSCs) affect the pathological processes of ischemic stroke via multiple targets and multitemporal, including reducing inflammation, modulating immune function, inhibiting apoptosis, promoting neurovascular regeneration, enhancing autophagy, and more (Zhou et al., 2022; Szydlak, 2023; Xie et al., 2023; Xu et al., 2023). A recent study indicated neuroprotective effects of bone MSCs transplantation, including reducing infarct size, improving motor function and behavioral outcomes, and downregulating NLRP3 inflammasome expression. However, all these positive effects were reversed by the Sirt1 specific inhibitor EX-527 through the regulation of NF-κB pathway (Sarmah et al., 2022). During the hyperacute phase of ischemic stroke, researchers observed the suppression of Sirt1 and upregulation of TRFA6 protein and ROS levels were observed. Activation of Sirt1 exerted its neuroprotection by inhibiting cellular pyroptosis after stroke via the ROS-TRFA6 signaling pathway (Yan et al., 2020).

Resveratrol, a specific Sirt1 agonist, performed the positive effect on the inhibition of NLRP3 inflammasome and neuroprotection after embolic stroke. Furthermore, it attenuated I/R-induced NLRP3 inflammasome-derived inflammation and upregulated autophagy. Sirt1 knockdown significantly blocked resveratrol-induced enhancement of autophagy activity and suppression of NLRP3 inflammasome activation, which implied that resveratrol protects against cerebral I/R injury by inhibiting NLRP3 inflammasome activation through Sirt1-dependent autophagy activity (He et al., 2017).

Recently, several studies indicated that acetylation of NLRP3 is required for the assembly and activation of the NLRP3 inflammasome (Zhao et al., 2019). So, suppressing acetylation of NLRP3 can inhibit the incidence and development of pyroptosis. Zhang et al. demonstrated that Sirt2 improved aging-associated chronic inflammation and insulin resistance by promoting NLRP3 deacetylation (He et al., 2020). Moreover, the inhibitory effect of Sirt1 on NLRP3 acetylation was also found in adipose tissue inflammation (Chen C. et al., 2022). Although various inhibitory mechanisms of Sirt1 on NLRP3 inflammasome have been discussed, whether Sirt1 exerts a protective effect on ischemic stroke by directly regulating NLRP3 deacetylation to inhibit pyroptosis remains unknown.

### Autophagy

Autophagy-dependent death, known as type 2 programmed cell death, is essential for maintaining cellular homeostasis in both physiological and pathological processes (Debnath et al., 2023). However, it is still unclear whether it has a positive or negative impact. Generally, in the nervous system, moderate autophagy has neuroprotective effects, while inadequate or excess autophagy may lead to neuronal death. Recently, autophagy has been recognized as a critical process in ischemic stroke in addition to neurodegenerative diseases (Yang Z. et al., 2022). And growing evidence suggests that Sirt1 may promote neuronal cell survival and alleviate cerebral I/R injury by modulating autophagy process (Tang et al., 2022; Teertam and Phanithi, 2022).

Nicotinamide phosphoribosyltransferase (Nampt), the rate-limiting enzyme in mammalian NAD^+^ biosynthesis, has been found to have a positive effect on ischemic stroke treatment. Besides inhibiting neuronal apoptosis and necrosis, Nampt promotes neuronal survival through inducing autophagy via regulating TSC2-mTOR-S6K1 signaling pathway in a Sirt1-dependent manner during cerebral ischemia (Wang et al., 2012). Nicotinamide mononucleotide adenylyltransferase also showed the similar therapeutic potential as Nampt for cerebral ischemia. It was reported that Nicotinamide mononucleotide adenylyltransferase protects against acute ischemic stroke in aged rats by inducing autophagy via regulating the Sirt1/mTOR pathway (Wang P. et al., 2019).

Electroacupuncture (EA) treatment is a promising therapy for ischemic stroke, however, the specific mechanism is still elusive. It is recently reported that EA treatment may inhibit apoptosis by regulating autophagy in the acute phase of ischemic stroke, thereby alleviating brain injury, and Sirt1 may play a crucial role in the regulation of autophagy in EA treatment for ischemic stroke (Xing et al., 2021). Xu et al. (2020) further tested the role of Sirt1 on regulating autophagy after ischemic stroke. They found that EA treatment inhibited the histone H4K16 acetylation process through Sirt1, facilitated autophagy, and alleviated I/R injury.

Diabetic brains are more vulnerable to I/R injury, but melatonin treatment has been found to protect against cerebral I/R-induced brain damage in both normal and diabetic mice by enhancing autophagy through the Sirt1-BMAL1 pathway (Liu L. et al., 2021).

Cerebral I/R injury induced by hemorrhagic shock and reperfusion is the main cause of death following trauma. Sirt1 was involved in the neuroprotective effects of sevoflurane post-conditioning on regulation of defective autophagy, mitochondrial oxidative injury, and neuronal death caused by hemorrhagic shock and reperfusion (Shu et al., 2022).

In addition to indirect regulation, Sirt1 can also directly modulate the deacetylation of the autophagy-related protein to induce autophagy. LC3, a key initiator of autophagy, became selectively activated in the nucleus during starvation through deacetylation by Sirt1. Deacetylation of LC3 at K49 and K51 by Sirt1 allows LC3 to interact with the nuclear protein DOR and return to the cytoplasm where it functioned as autophagy initiation (Huang et al., 2015). Recently reported, deacetylation of beclin1 was also mediated by Sirt1, which improved the acute kidney injury via activation of autophagy (Deng et al., 2021). However, the direct regulatory effect of Sirt1 on autophagy in stroke remains unclear and needs to be confirmed by further studies.

Interestingly, Sirt1 not only can induce autophagy after ischemic stroke, but also has a negative regulatory effect on autophagy.

The activation of Sirt1/FOXO1 pathway by Betulinic acid, a pentacyclic triterpene acid mainly extracted from birch bark, suppressed the autophagy, which improved the brain damage after ischemic stroke (Zhao et al., 2021). Magnoflorine, a natural compound with anti-oxidant and immunomodulatory effects, has also been found to protect against ischemic stroke by inhibiting autophagy through the activation of the Sirt1/AMPK pathway (Liang et al., 2022).

As mentioned above, there is bidirectional regulation of autophagy by Sirt1 in stroke. Further studies are needed to thoroughly understand the regulatory effect of Sirt1 on autophagy, which is of great significance for the prevention and treatment of stroke.

### Necroptosis

Necroptosis is a programmed type of cell death mediated by receptor-interacting serine/threonine-protein kinase (RIPK) 1, RIPK3, and mixed lineage kinase like protein (MLKL), which is characterized by cellular organelle swelling and cell membrane rupture (Albani et al., 2010). This process plays a critical role in both physiological and pathological conditions, and Sirt1 has been shown to protect against necroptosis in various disease models, including cancer (Carafa et al., 2018), acute lung injury (Liu et al., 2022) and liver fibrosis (Sun et al., 2022). However, a study of ischemic stroke has yielded contradictory results. Specifically, RIP3 and MLKL levels were found to increase in the prefrontal cortex and hippocampus of rat brains during the 24 h after I/R injury.

Surprisingly, the Sirt1 inhibitor EX-527 was shown to be as effective as necrostatin-1 in suppressing the elevation of RIP3 and MLKL, leading to reduced infarct volumes, which indicated that suppression of Sirt1 provided the neuroprotection against ischemic stroke by inhibiting necroptosis. Further studies are needed to elucidate the interaction between Sirt1 and necroptosis following ischemic stroke.

### Ferroptosis

Ferroptosis is a form of programmed cell death that depends on iron overload and lipid peroxidation, and has gained significant attention since its discovery in Lei et al. (2022). Excessive intracellular iron accumulation results in the production of reactive oxygen species (ROS) through the Fenton reaction, causing lipid peroxidation and subsequent ferroptosis. Studies have shown that iron deposition, lipid peroxidation, and neuronal death in the brain were significantly increased in an adult rat model of ischemic stroke (Ye et al., 2022).

Glutathione peroxidase 4 (GPX4) plays an important role in suppressing ferroptosis, which functions to reduce lipid peroxides in cellular membranes.

Silent mating type information regulation 2 homolog 1 activator resveratrol exhibited the positive effects on inhibiting ferroptosis via upregulation of GPX4, which exerted neuroprotection against ischemic stroke. Our previous research found that resveratrol pretreatment had a similar effect as ferroptosis inhibitors, ferrostatin-1 on inhibiting neuronal ferroptosis-related changes, such as iron overload, damages of oxidation-reduction system, and destruction of mitochondrial structure, with the upregulation of GPX4 (Zhu et al., 2022). Similarly, Li C. et al. (2022) found that resveratrol inhibited hippocampal neuronal ferroptosis by activating Sirt1/Nrf2/GPx4 signaling pathway, thereby improving the cognitive impairment.

Furthermore, it was recently demonstrated that Sirt1 participated in the neuroprotection against ischemic stroke both *in vivo* and *in vitro* by inhibiting ferroptosis via SLC7A11, another key executor of ferroptosis. Further researches are needed to determine whether Sirt1 can inhibit ferroptosis by directly regulating the deacetylation of ferroptosis-associated molecules and thus exert neuroprotective effects.

### Cuproptosis

Copper is an indispensable cofactor for all organisms, but excessive intracellular copper induces cell death, thus causing toxic effects on the body. Recently, Todd R. Golub and Peter Tsvetkov et al. (2022) found a new sort of copper-dependent programmed cell death, and named it cuproptosis. Cuproptosis occurs through direct interaction of copper with the fatty acylated components of the tricarboxylic acid cycle, leading to excessive aggregation of fatty acylated proteins and loss of iron–sulfur cluster proteins, which stimulates proteotoxic stress and cell death. In ischemic stroke patients, the level of copper in serum and urine was significantly increased (Lai et al., 2016). However, it requires further to be clarification whether excessive copper induces cuproptosis of neurons and whether Sirt1 played a key role in regulating cuproptosis.

### Promoting angiogenesis

Angiogenesis can promote the survival and recovery of patients with ischemic stroke by restoring blood supply to the affected regions. Emerging evidence has indicated the involvement of Sirt1 in post-stroke angiogenesis, which is a complicated process regulated by angiogenic factors, such as vascular endothelial growth factor (VEGF) (Simão et al., 2012; Hermann et al., 2015; Zheng et al., 2018).

Hypoxia inducible factor 1α (HIF-1α) is the core regulatory factor of post-stroke angiogenesis, which can upregulate the expression of key angiogenic factors, such as VEGF and its receptor, thereby promoting post-stroke angiogenesis. The interaction between Sirt1 and HIF-1α was first reported in Lim et al. (2010). It revealed that Sirt1 could interact with HIF-1α and deacetylate its 647 lysine to inhibit its activity and thereby suppressing angiogenesis. Conversely, a recent study has indicated that Sirt1 can promote the proliferation and migration of hypoxia/high glucose induced-BMECs by activating HIF-1α/VEGF pathway, which is the important process of angiogenesis (Mi et al., 2019). Further studies are needed to clarify the relationship between Sirt1 and HIF-1α-mediated angiogenesis.

Vascular endothelial growth factor acts directly on endothelial cells and is a critical node in the angiogenic process. Choi et al. (2017) found that Sirt1 could upregulate the expression of VEGF through inducing PGC-1α deacetylation and ubiquitination to promote angiogenesis. Furthermore, Donepezil was reported to increase the viability and migration of OGD/R-induced human BMECs and expression of VEGF via Sirt1/FOXO3a/NF-κB pathway (Sun and Liu, 2022). The Notch signaling pathway and VEGF exhibit a synergistic effect in angiogenesis, especially in the process of tube formation (Gerhardt et al., 2003). Notoginsenoside R1, a natural constituent, could promote angiogenesis via Notch/VEGF signaling pathway, which was partially reversed by Sirt1 inhibitor EX527 (Zhu et al., 2021). However, it remains unclear how Sirt1 regulates the interaction between Notch signaling and VEGF to promote angiogenesis in ischemic stroke.

### Enhancing neurogenesis

Neurogenesis, which involves the proliferation and differentiation of neural stem cells (NSCs), is crucial for functional recovery after ischemic stroke. Sirt1 has shown the potential property of inducing neurogenesis primarily through sonic hedgehog (Shh) signaling and Wnt/β-catenin signaling.

It was found that up-regulation of Sirt1 activity by momordica charantia polysaccharides induced the cytoplasmic deacetylation of β-catenin, which mediated the translocation of β-catenin into the nucleus, thus promoting NSCs proliferation in the subventricular and subgranular zones of cerebral I/R rats on the one hand (Ma et al., 2021), and transferring the differentiation potential of NSCs from the gliogenic to neurogenic lineage under pathological conditions on the other hand (Hu et al., 2020). Taken together, Sirt1 can induce neurogenesis, including NSCs proliferation and differentiation, thereby promoting recovery from cerebral I/R injury.

Sonic hedgehog signaling plays a critical role in regulating stem cell behavior and promoting neurite outgrowth and synaptogenesis in both developing and adult brains. Our previous studies suggested that Sirt1 activator resveratrol pretreatment enhanced NSCs proliferation *in vitro* (Cheng et al., 2015) and *in vivo* (Yu H. et al., 2021) after cerebral I/R injury, and induced the differentiation of bone MSCs into neuronal-like cells via activation of the Shh signaling (Huang et al., 2014).

In conclusion, Sirt1 has shown its protective effects on endogenous NSCs proliferation and differentiation. However, further studies are necessary to clarify whether Sirt1 contributes to the survival of exogenous stem cell transplantation.

## Conclusion and perspectives

Ischemic stroke has long caused concern among medical professionals as one of the leading causes of death worldwide. Therefore, the need for novel treatment modalities is urgent at the moment. The data we gathered has identified activated Sirt1 as a potential therapy. It is clear that Sirt1 is able to protect against pathological situations like cerebral ischemia injury and sustain brain homeostasis when acting physiologically. Numerous pharmacological agents that stimulate Sirt1 have been thoroughly described above and have demonstrated the potential for clinical transformation. However, despite these encouraging findings, there is still a lack of clinical proof to support the claim that Sirt1 protects against ischemia stroke. Additional research is needed to substantiate this claim.

The specific mechanism by which Sirt1 promotes neuroprotection in ischemic stroke is not yet completely clear. Therefore, further investigation is required to identify the precise target of Sirt1, which will aid in the development of novel treatment strategies for ischemic stroke. In summary, Sirt1 is undoubtedly a promising candidate therapeutic target for ischemic stroke.

## Author contributions

HT, JW, and TQ: data curation. HT, JW, TQ, and YC: framework design. HT: writing and original draft preparation. JH, QhY, and PJ: language and format revision. QiY: review and editing. HT, LW, and YZ: revision and supervision. All authors contributed to the article and approved the submitted version.

## References

[B1] Ahmadi M. Laumeier I. Ihl T. Steinicke M. Ferse C. Endres M. (2020). A support programme for secondary prevention in patients with transient ischaemic attack and minor stroke (INSPiRE-TMS): An open-label, randomised controlled trial. *Lancet Neurol.* 19 49–60. 10.1016/S1474-4422(19)30369-2 31708447

[B2] Al-Bahrani R. Tuertcher D. Zailaie S. Abuetabh Y. Nagamori S. Zetouni N. (2015). Differential SIRT1 expression in hepatocellular carcinomas and cholangiocarcinoma of the liver. *Ann Clin. Lab. Sci.* 45 3–9. 25696003

[B3] Albani D. Polito L. Signorini A. Forloni G. (2010). Neuroprotective properties of resveratrol in different neurodegenerative disorders. *BioFactors* 36 370–376. 10.1002/biof.118 20848560

[B4] Autiero I. Costantini S. Colonna G. (2008). Human sirt-1: Molecular modeling and structure-function relationships of an unordered protein. *PLoS One* 4:e7350. 10.1371/journal.pone.0007350 19806227 PMC2753774

[B5] Bai L. Liu R. Wang R. Xin Y. Wu Z. Ba Y. (2021). Attenuation of Pb-induced Aβ generation and autophagic dysfunction via activation of SIRT1: Neuroprotective properties of resveratrol. *Ecotoxicol. Environ. Safety* 222:112511. 10.1016/j.ecoenv.2021.112511 34273848

[B6] Cao D. Wang M. Qiu X. Liu D. Jiang H. Yang N. (2015). Structural basis for allosteric, substrate-dependent stimulation of SIRT1 activity by resveratrol. *Genes Dev.* 29 1316–1325. 10.1101/gad.265462.115 26109052 PMC4495401

[B7] Cao K. Dong Y. Xiang J. Xu Y. Hong W. Song H. (2018). Reduced expression of SIRT1 and SOD-1 and the correlation between these levels in various regions of the brains of patients with Alzheimer’s disease. *J. Clin. Pathol.* 71 1090–1099. 10.1136/jclinpath-2018-205320 30185534

[B8] Carafa V. Nebbioso A. Cuomo F. Rotili D. Cobellis G. Bontempo P. (2018). RIP1-HAT1-SIRT complex identification and targeting in treatment and prevention of cancer. *Clin. Cancer Res.* 24 2886–2900. 10.1158/1078-0432.CCR-17-3081 29535128

[B9] Carneiro B. El-Deiry W. (2020). Targeting apoptosis in cancer therapy. *Nat. Rev. Clin. Oncol.* 17 395–417. 10.1038/s41571-020-0341-y 32203277 PMC8211386

[B10] Chang H. Guarente L. (2014). SIRT1 and other sirtuins in metabolism. *Trends Endocrinol. Metab.* 25 138–145. 10.1016/j.tem.2013.12.001 24388149 PMC3943707

[B11] Chen C. Ren Y. Zhu J. Chen J. Feng Z. Zhang T. (2022). Ainsliadimer C, a disesquiterpenoid isolated from Ainsliaea macrocephala, ameliorates inflammatory responses in adipose tissue via sirtuin 1-NLRP3 inflammasome axis. *Acta Pharmacol. Sin.* 43 1780–1792. 10.1038/s41401-021-00797-z 34789920 PMC9253034

[B12] Chen G. Shan X. Li L. Dong L. Huang G. Tao H. (2022). circHIPK3 regulates apoptosis and mitochondrial dysfunction induced by ischemic stroke in mice by sponging miR-148b-3p via CDK5R1/SIRT1. *Exp. Neurol.* 355:114115. 10.1016/j.expneurol.2022.114115 35576990

[B13] Chen J. Bai Q. Zhao Z. Sui H. Xie X. (2016). Resveratrol improves delayed r-tPA treatment outcome by reducing MMPs. *Acta Neurol. Scand.* 134 54–60. 10.1111/ane.12511 26455907

[B14] Cheng W. Yu P. Wang L. Shen C. Song X. Chen J. (2015). Sonic hedgehog signaling mediates resveratrol to increase proliferation of neural stem cells after oxygen-glucose deprivation/reoxygenation injury in vitro. *Cell. Physiol. Biochem.* 35 2019–2032. 10.1159/000374009 25871875

[B15] Chiang M. Nicol C. Lo S. Hung S. Wang C. Lin C. (2022). Resveratrol mitigates oxygen and glucose deprivation-induced inflammation, NLRP3 inflammasome, and oxidative stress in 3D neuronal culture. *Int. J. Mol. Sci.* 23:11678. 10.3390/ijms231911678 36232980 PMC9570351

[B16] Choi Y. Kim J. Lee D. Lee K. Won M. Jeoung D. (2017). Carbon monoxide potentiation of L-Type Ca channel activity increases HIF-1α-independent VEGF expression via an AMPKα/SIRT1-Mediated PGC-1α/ERRα Axis. *Antioxid. Redox Signal.* 27 21–36. 10.1089/ars.2016.6684 27554679

[B17] Cui Z. Zhao X. Amevor F. Du X. Wang Y. Li D. (2022). Therapeutic application of quercetin in aging-related diseases: SIRT1 as a potential mechanism. *Front. Immunol.* 13:943321. 10.3389/fimmu.2022.943321 35935939 PMC9355713

[B18] Debnath J. Gammoh N. Ryan K. (2023). Autophagy and autophagy-related pathways in cancer. *Nat. Rev. Mol. Cell biol.* 24 560–575. 10.1038/s41580-023-00585-z 36864290 PMC9980873

[B19] Deng Z. Sun M. Wu J. Fang H. Cai S. An S. (2021). SIRT1 attenuates sepsis-induced acute kidney injury via beclin1 deacetylation-mediated autophagy activation. *Cell Death Dis.* 12:217. 10.1038/s41419-021-03508-y 33637691 PMC7910451

[B20] Di Leva G. Garofalo M. Croce C. (2014). MicroRNAs in cancer. *Annu. Rev. Pathol.* 9 287–314. 10.1093/neuonc/noz215 24079833 PMC4009396

[B21] Esmayel I. Hussein S. Gohar E. Ebian H. Mousa M. (2021). Plasma levels of sirtuin-1 in patients with cerebrovascular stroke. *Neurol. Sci.* 42 3843–3850. 10.1007/s10072-021-05074-9 33507417

[B22] Fodor K. Tit D. Pasca B. Bustea C. Uivarosan D. Endres L. (2018). Long-term resveratrol supplementation as a secondary prophylaxis for stroke. *Oxid. Med. Cell. Longev.* 2018:4147320. 10.1155/2018/4147320 29743980 PMC5878880

[B23] Frye R. (1999). Characterization of five human cDNAs with homology to the yeast SIR2 gene: Sir2-like proteins (sirtuins) metabolize NAD and may have protein ADP-ribosyltransferase activity. *Biochem. Biophys. Res. Commun.* 260 273–279. 10.1006/bbrc.1999.0897 10381378

[B24] Fu C. Zhong C. Yang Y. Zhang M. Li W. Zhou Q. (2021). Sirt1 activator SRT2104 protects against oxygen-glucose deprivation/reoxygenation-induced injury via regulating microglia polarization by modulating Sirt1/NF-κB pathway. *Brain Res.* 1753:147236. 10.1016/j.brainres.2020.147236 33412146

[B25] Gao H. Yang L. Shao Y. (2022). SIRT1/NF-κB pathway on neuronal apoptosis in rats with ischemic stroke. *Cell. Mol. Biol.* 68 77–82. 10.26355/eurrev_201906_18214 36029487

[B26] Gerhardt H. Golding M. Fruttiger M. Ruhrberg C. Lundkvist A. Abramsson A. (2003). VEGF guides angiogenic sprouting utilizing endothelial tip cell filopodia. *J Cell Biol.* 161 1163–1177. 10.1083/jcb.200302047 12810700 PMC2172999

[B27] Gertz M. Fischer F. Nguyen G. Lakshminarasimhan M. Schutkowski M. Weyand M. (2013). Ex-527 inhibits sirtuins by exploiting their unique NAD+-dependent deacetylation mechanism. *Proc. Natl. Acad. Sci. U. S. A.* 110 E2772–E2781. 10.1073/pnas.1303628110 23840057 PMC3725051

[B28] Hattori Y. Okamoto Y. Nagatsuka K. Takahashi R. Kalaria R. Kinoshita M. (2015). SIRT1 attenuates severe ischemic damage by preserving cerebral blood flow. *Neuroreport* 26 113–117. 10.1097/WNR.0000000000000308 25634315

[B29] He M. Chiang H. Luo H. Zheng Z. Qiao Q. Wang L. (2020). An acetylation switch of the NLRP3 inflammasome regulates aging-associated chronic inflammation and insulin resistance. *Cell Metab.* 31 580–591.e5. 10.1016/j.cmet.2020.01.009 32032542 PMC7104778

[B30] He Q. Li Z. Wang Y. Hou Y. Li L. Zhao J. (2017). Resveratrol alleviates cerebral ischemia/reperfusion injury in rats by inhibiting NLRP3 inflammasome activation through Sirt1-dependent autophagy induction. *Int. Immunopharmacol.* 50 208–215. 10.1016/j.intimp.2017.06.029 28683365

[B31] Heinisch O. Zeyen T. Goldmann T. Prinz M. Huber M. Jung J. (2022). Erythropoietin abrogates post-ischemic activation of the NLRP3, NLRC4, and aim2 inflammasomes in microglia/macrophages in a TAK1-dependent manner. *Transl. Stroke Res.* 13 462–482. 10.1007/s12975-021-00948-8 34628598 PMC9046144

[B32] Hermann D. Zechariah A. Kaltwasser B. Bosche B. Caglayan A. Kilic E. (2015). Sustained neurological recovery induced by resveratrol is associated with angioneurogenesis rather than neuroprotection after focal cerebral ischemia. *Neurobiol. Dis.* 83 16–25. 10.1016/j.nbd.2015.08.018 26316359

[B33] Hernández-Jiménez M. Hurtado O. Cuartero M. Ballesteros I. Moraga A. Pradillo J. (2013). Silent information regulator 1 protects the brain against cerebral ischemic damage. *Stroke* 44 2333–2337. 10.1161/STROKEAHA.113.001715 23723308

[B34] Herskovits A. Guarente L. (2014). SIRT1 in neurodevelopment and brain senescence. *Neuron* 81 471–483. 10.1016/j.neuron.2014.01.028 24507186 PMC4040287

[B35] Hisahara S. Chiba S. Matsumoto H. Tanno M. Yagi H. Shimohama S. (2008). Histone deacetylase SIRT1 modulates neuronal differentiation by its nuclear translocation. *Proc. Natl. Acad. Sci. U. S. A.* 105 15599–15604. 10.1073/pnas.0800612105 18829436 PMC2563076

[B36] Hu C. Zhang S. Chen Q. Wang R. (2022). Ovatodiolide protects ischemia-reperfusion-induced neuronal injury via microglial neuroinflammation via mediating SIRT1/NF-κB pathway. *Brain Res. Bull.* 180 97–107. 10.1016/j.brainresbull.2021.12.010 34968641

[B37] Hu Z. Li F. Zhou X. Zhang F. Huang L. Gu B. (2020). *Momordica charantia* polysaccharides modulate the differentiation of neural stem cells via SIRT1/B -catenin axis in cerebral ischemia/reperfusion. *Stem Cell Res. Therapy* 11:485. 10.1186/s13287-020-02000-2 33198798 PMC7667795

[B38] Huang J. Shen C. Wu W. Ren J. Xu L. Liu S. (2014). Primary cilia mediate sonic hedgehog signaling to regulate neuronal-like differentiation of bone mesenchymal stem cells for resveratrol induction in vitro. *J. Neurosci. Res.* 92 587–596. 10.1002/jnr.23343 24464877

[B39] Huang R. Xu Y. Wan W. Shou X. Qian J. You Z. (2015). Deacetylation of nuclear LC3 drives autophagy initiation under starvation. *Mol. Cell* 57 456–466. 10.1016/j.molcel.2014.12.013 25601754

[B40] Jia N. Sun Q. Su Q. Chen G. (2016). SIRT1-mediated deacetylation of PGC1α attributes to the protection of curcumin against glutamate excitotoxicity in cortical neurons. *Biochem. Biophys. Res. Commun.* 478 1376–1381. 27568287 10.1016/j.bbrc.2016.08.132

[B41] Jin Q. Yan T. Ge X. Sun C. Shi X. Zhai Q. (2007). Cytoplasm-localized SIRT1 enhances apoptosis. *J. Cell. Physiol.* 213 88–97. 10.1002/jcp.21091 17516504

[B42] Kalaivani P. Ganesh M. Sathiya S. Ranju V. Gayathiri V. Saravana Babu C. (2014). Alteration in bioenergetic regulators, SirT1 and Parp1 expression precedes oxidative stress in rats subjected to transient cerebral focal ischemia: Molecular and histopathologic evidences. *J. Stroke Cerebrovasc. Dis.* 23 2753–2766. 10.1016/j.jstrokecerebrovasdis.2014.06.026 25440363

[B43] Kannan V. Brouwer N. Hanisch U. Regen T. Eggen B. Boddeke H. (2013). Histone deacetylase inhibitors suppress immune activation in primary mouse microglia. *J. Neurosci. Res.* 91 1133–1142. 10.1002/jnr.23221 23686642

[B44] Kerr N. Sanchez J. O’Connor G. Watson B. Daunert S. Bramlett H. (2022). Inflammasome-regulated pyroptotic cell death in disruption of the gut-brain axis after stroke. *Transl. Stroke Res.* 13 898–912. 35306629 10.1007/s12975-022-01005-8

[B45] Kim E. J. Kho J. H. Kang M. R. Um S. J. (2007). Active regulator of SIRT1 cooperates with SIRT1 and facilitates suppression of p53 activity. *Mol. Cell* 28 277–290. 10.1016/j.molcel.2007.08.030 17964266

[B46] Kim J. Chen J. Lou Z. (2008). DBC1 is a negative regulator of SIRT1. *Nature* 451 583–586. 18235501 10.1038/nature06500

[B47] Knight J. Allison S. Milner J. (2013). Active regulator of SIRT1 is required for cancer cell survival but not for SIRT1 activity. *Open Biol.* 3:130130. 10.1098/rsob.130130 24258275 PMC3843821

[B48] Lai M. Wang D. Lin Z. Zhang Y. (2016). Small molecule copper and its relative metabolites in serum of cerebral ischemic stroke patients. *J. Stroke cerebrovasc. Dis.* 25 214–219. 10.1016/j.jstrokecerebrovasdis.2015.09.020 26573522

[B49] Lanzillotta A. Sarnico I. Ingrassia R. Boroni F. Branca C. Benarese M. (2010). The acetylation of RelA in Lys310 dictates the NF-κB-dependent response in post-ischemic injury. *Cell Death Dis.* 1:e96. 10.1038/cddis.2010.76 PMC3032326 21368872

[B50] Lazo-Gomez R. Tapia R. (2017). Quercetin prevents spinal motor neuron degeneration induced by chronic excitotoxic stimulus by a sirtuin 1-dependent mechanism. *Transl. Neurodegen.* 6:31. 10.1186/s40035-017-0102-8 29201361 PMC5697078

[B51] Le K. Chibaatar Daliv E. Wu S. Qian F. Ali A. Yu D. (2019). SIRT1-regulated HMGB1 release is partially involved in TLR4 signal transduction: A possible anti-neuroinflammatory mechanism of resveratrol in neonatal hypoxic-ischemic brain injury. *Int. Immunopharmacol.* 75:105779. 10.1016/j.intimp.2019.105779 31362164

[B52] Lei G. Zhuang L. Gan B. (2022). Targeting ferroptosis as a vulnerability in cancer. *Nature Rev. Cancer* 22 381–396. 35338310 10.1038/s41568-022-00459-0 PMC10243716

[B53] Li C. Wu Z. Xue H. Gao Q. Zhang Y. Wang C. (2022). Ferroptosis contributes to hypoxic-ischemic brain injury in neonatal rats: Role of the SIRT1/Nrf2/GPx4 signaling pathway. *CNS Neurosci. Therap.* 28 2268–2280. 10.1111/cns.13973 36184790 PMC9627393

[B54] Li H. Li X. Hao Y. Wu C. Fu Y. Su N. (2022). Maresin 1 intervention reverses experimental pulmonary arterial hypertension in mice. *Br. J. Pharmacol.* 179 5132–5147. 10.1111/bph.15906 35764296

[B55] Li J. Zhang Z. Wang L. Jiang L. Qin Z. Zhao Y. (2021). Maresin 1 Attenuates Lipopolysaccharide-Induced Acute Kidney Injury via Inhibiting NOX4/ROS/NF-κB Pathway. *Front. Pharmacol.* 12:782660. 10.3389/fphar.2021.782660 34955852 PMC8703041

[B56] Liang H. Chang X. Xia R. Wu W. Guo H. Yang M. (2022). Magnoflorine attenuates cerebral ischemia-induced neuronal injury via autophagy/Sirt1/AMPK signaling pathway. *Evid. Based Complem. Altern. Med.* 2022:2131561. 10.1155/2022/2131561 36124014 PMC9482485

[B57] Lim J. Lee Y. Chun Y. Chen J. Kim J. Park J. (2010). Sirtuin 1 modulates cellular responses to hypoxia by deacetylating hypoxia-inducible factor 1alpha. *Mol. Cell* 38 864–878. 10.1016/j.molcel.2010.05.023 20620956

[B58] Ling C. Liang J. Zhang C. Li R. Mou Q. Qin J. (2018). Synergistic effects of salvianolic acid B and puerarin on cerebral ischemia reperfusion injury. *Molecules* 23:564. 10.3390/molecules23030564 29498696 PMC6017479

[B59] Liu B. Ghosh S. Yang X. Zheng H. Liu X. Wang Z. (2012). Resveratrol rescues SIRT1-dependent adult stem cell decline and alleviates progeroid features in laminopathy-based progeria. *Cell Met.* 16 738–750. 10.1016/j.cmet.2012.11.007 23217256

[B60] Liu L. Cao Q. Gao W. Li B. Zeng C. Xia Z. (2021). Melatonin ameliorates cerebral ischemia-reperfusion injury in diabetic mice by enhancing autophagy via the SIRT1-BMAL1 pathway. *FASEB J.* 35:e22040. 10.1096/fj.202002718RR 34800293

[B61] Liu R. Li H. Deng J. Wu Q. Liao C. Xiao Q. (2021). QKI 6 ameliorates CIRI through promoting synthesis of triglyceride in neuron and inhibiting neuronal apoptosis associated with SIRT1-PPARγ-PGC-1α axis. *Brain Behav.* 11:e2271. 10.1002/brb3.2271 PMC8413718 34227244

[B62] Liu Y. Jia S. Liang X. Dong M. Xu X. Lu C. (2018). Prognostic value of Sirtuin1 in acute ischemic stroke and its correlation with functional outcomes. *Medicine* 97:e12959. 10.1097/MD.0000000000012959 30544370 PMC6310560

[B63] Liu Z. Li C. Li Y. Yu L. Qu M. (2022). Propofol reduces renal ischemia reperfusion-mediated necroptosis by up-regulation of SIRT1 in rats. *Inflammation* 45 2038–2051. 10.1007/s10753-022-01673-6 35460396

[B64] Lv H. Wang L. Shen J. Hao S. Ming A. Wang X. (2015). Salvianolic acid B attenuates apoptosis and inflammation via SIRT1 activation in experimental stroke rats. *Brain Res. Bull.* 115 30–36. 10.1016/j.brainresbull.2015.05.002 25981395

[B65] Ma J. Fan H. Cai H. Hu Z. Zhou X. Li F. (2021). Promotion of *Momordica Charantia* polysaccharides on neural stem cell proliferation by increasing SIRT1 activity after cerebral ischemia/reperfusion in rats. *Brain Res. Bull.* 170 254–263. 10.1016/j.brainresbull.2021.02.016 33647420

[B66] Meng F. Qian M. Peng B. Peng L. Wang X. Zheng K. (2020). Synergy between SIRT1 and SIRT6 helps recognize DNA breaks and potentiates the DNA damage response and repair in humans and mice. *eLife* 9:e55828. 10.7554/eLife.55828 32538779 PMC7324161

[B67] Meng S. Wang B. Li W. (2023). LncRNA MALAT1 improves cerebral ischemia-reperfusion injury and cognitive dysfunction by regulating miR-142-3p/SIRT1 axis. *Int J Neurosci.* 133 740–753. 10.1080/00207454.2021.1972999 34461809

[B68] Mi D. Fang H. Zheng G. Liang X. Ding Y. Liu X. (2019). DPP-4 inhibitors promote proliferation and migration of rat brain microvascular endothelial cells under hypoxic/high-glucose conditions, potentially through the SIRT1/HIF-1/VEGF pathway. *CNS Neurosci. Therap.* 25 323–332. 10.1111/cns.13042 30136405 PMC6488877

[B69] Milne J. Lambert P. Schenk S. Carney D. Smith J. Gagne D. (2007). Small molecule activators of SIRT1 as therapeutics for the treatment of type 2 diabetes. *Nature* 450 712–716. 18046409 10.1038/nature06261 PMC2753457

[B70] Nogueira R. Jadhav A. Haussen D. Bonafe A. Budzik R. Bhuva P. (2018). Thrombectomy 6 to 24 hours after stroke with a mismatch between deficit and infarct. *N. Engl. J. Med.* 378 11–21. 29129157 10.1056/NEJMoa1706442

[B71] Ogawa T. Wakai C. Saito T. Murayama A. Mimura Y. Youfu S. (2011). Distribution of the longevity gene product. SIRT1, in developing mouse organs. *Congenital Anom.* 51 70–79. 10.1111/j.1741-4520.2010.00304.x 21054562

[B72] Pérez S. Rius-Pérez S. Finamor I. Martí-Andrés P. Prieto I. García R. (2019). Obesity causes PGC-1α deficiency in the pancreas leading to marked IL-6 upregulation via NF-κB in acute pancreatitis. *J. Pathol.* 247 48–59. 30221360 10.1002/path.5166

[B73] Prozorovski T. Ingwersen J. Lukas D. Göttle P. Koop B. Graf J. (2019). Regulation of sirtuin expression in autoimmune neuroinflammation: Induction of SIRT1 in oligodendrocyte progenitor cells. *Neurosci. Lett.* 704 116–125. 10.1016/j.neulet.2019.04.007 30953735

[B74] Ramadori G. Lee C. Bookout A. Lee S. Williams K. Anderson J. (2008). Brain SIRT1: Anatomical distribution and regulation by energy availability. *J. Neurosci.* 28 9989–9996. 10.1523/JNEUROSCI.3257-08.2008 18829956 PMC2578850

[B75] Rana P. Franco E. Rao Y. Syed K. Barh D. Azevedo V. (2019). Evaluation of the common molecular basis in Alzheimer’s and Parkinson’s diseases. *Int. J. Mol. Sci.* 20:3730. 10.3390/ijms20153730 PMC6695669 31366155

[B76] Sakamoto J. Miura T. Shimamoto K. Horio Y. (2004). Predominant expression of Sir2alpha, an NAD-dependent histone deacetylase, in the embryonic mouse heart and brain. *FEBS Lett.* 556 281–286. 10.1016/s0014-5793(03)01444-3 14706864

[B77] Sarmah D. Datta A. Kaur H. Kalia K. Borah A. Rodriguez A. (2022). Sirtuin-1 – mediated NF-κB pathway modulation to mitigate inflammasome signaling and cellular apoptosis is one of the neuroprotective effects of intra-arterial mesenchymal stem cell therapy following ischemic stroke. *Stem Cell Rev. Rep.* 18 821–838. 35112234 10.1007/s12015-021-10315-7

[B78] Sauve A. Wolberger C. Schramm V. Boeke J. (2006). The biochemistry of sirtuins. *Annu. Rev. Biochem.* 75 435–465. 16756498 10.1146/annurev.biochem.74.082803.133500

[B79] Sharma B. Kanneganti T. (2021). NLRP3 inflammasome in cancer and metabolic diseases. *Nat. Immunol.* 22 550–559. 33707781 10.1038/s41590-021-00886-5 PMC8132572

[B80] Shen C. Cheng W. Yu P. Wang L. Zhou L. Zeng L. (2016). Resveratrol pretreatment attenuates injury and promotes proliferation of neural stem cells following oxygen-glucose deprivation/reoxygenation by upregulating the expression of Nrf2, HO-1 and NQO1 in vitro. *Mol. Med. Rep.* 14 3646–3654. 10.3892/mmr.2016.5670 27573874 PMC5042764

[B81] Shu J. Huang X. Liao Q. Wang J. Zhou Y. Chen Y. (2022). Sevoflurane improves hemorrhagic shock and resuscitation-induced cognitive impairments and mitochondrial dysfunctions through SIRT1-mediated autophagy. *Oxid. Med. Cell. Longev.* 2022:9771743. 10.1155/2022/9771743 35528522 PMC9068312

[B82] Simão F. Pagnussat A. Seo J. Navaratna D. Leung W. Lok J. (2012). Pro-angiogenic effects of resveratrol in brain endothelial cells: Nitric oxide-mediated regulation of vascular endothelial growth factor and metalloproteinases. *J. Cereb. Blood Flow Metab.* 32 884–895. 10.1038/jcbfm.2012.2 22314268 PMC3345913

[B83] Song L. Mu L. Wang H. (2022). MicroRNA-489-3p aggravates neuronal apoptosis and oxidative stress after cerebral ischemia-reperfusion injury. *Bioengineered* 13 14047–14056. 10.1080/21655979.2022.2062534 35730531 PMC9342425

[B84] St-Pierre J. Drori S. Uldry M. Silvaggi J. Rhee J. Jäger S. (2006). Suppression of reactive oxygen species and neurodegeneration by the PGC-1 transcriptional coactivators. *Cell* 127 397–408. 10.1016/j.cell.2006.09.024 17055439

[B85] Sun S. Li Z. Huan S. Kai J. Xia S. Su Y. (2022). Modification of lysine deacetylation regulates curcumol-induced necroptosis through autophagy in hepatic stellate cells. *Phytother. Res.* 36 2660–2676. 10.1002/ptr.7483 35545249

[B86] Sun X. Liu B. (2022). Donepezil ameliorates oxygen-glucose deprivation/reoxygenation-induced brain microvascular endothelial cell dysfunction via the SIRT1/FOXO3a/NF-κB pathways. *Bioengineered* 13 7760–7770. 35286233 10.1080/21655979.2022.2045833 PMC9208472

[B87] Szabo G. Bala S. (2013). MicroRNAs in liver disease. *Nat. Rev. Gastroenterol. hepatol.* 10 542–552. 23689081 10.1038/nrgastro.2013.87 PMC4091636

[B88] Szydlak R. (2023). Mesenchymal stem cells in ischemic tissue regeneration. *World J. Stem Cells* 15 16–30. 36909782 10.4252/wjsc.v15.i2.16 PMC9993139

[B89] Tang F. Guo S. Liao H. Yu P. Wang L. Song X. (2017). Resveratrol enhances neurite outgrowth and synaptogenesis via sonic hedgehog signaling following oxygen-glucose deprivation/reoxygenation injury. *Cell. Physiol. Biochem.* 43 852–869. 10.1159/000481611 28957797

[B90] Tang Y. Xie J. Chen X. Sun L. Xu L. Chen X. (2022). A novel link between silent information regulator 1 and autophagy in cerebral ischemia-reperfusion. *Front. Neurosci.* 16:1040182. 10.3389/fnins.2022.1040182 36507335 PMC9726917

[B91] Tanner K. Landry J. Sternglanz R. Denu J. (2000). Silent information regulator 2 family of NAD- dependent histone/protein deacetylases generates a unique product, 1-O-acetyl-ADP-ribose. *Proc. Natl. Acad. Sci. U. S. A.* 97 14178–14182. 11106374 10.1073/pnas.250422697 PMC18891

[B92] Tanno M. Kuno A. Yano T. Miura T. Hisahara S. Ishikawa S. (2010). Induction of manganese superoxide dismutase by nuclear translocation and activation of SIRT1 promotes cell survival in chronic heart failure. *J. Biol. Chem.* 285 8375–8382. 10.1074/jbc.M109.090266 20089851 PMC2832987

[B93] Tanno M. Sakamoto J. Miura T. Shimamoto K. Horio Y. (2007). Nucleocytoplasmic shuttling of the NAD+-dependent histone deacetylase SIRT1. *J. Biol. Chem.* 282 6823–6832. 17197703 10.1074/jbc.M609554200

[B94] Teertam S. Jha S. Prakash Babu P. (2020). Up-regulation of Sirt1/miR-149-5p signaling may play a role in resveratrol induced protection against ischemia via p53 in rat brain. *J. Clin. Neurosci.* 72 402–411. 10.1016/j.jocn.2019.11.043 31866350

[B95] Teertam S. Phanithi P. (2022). Up-regulation of Sirtuin-1/autophagy signaling in human cerebral ischemia: Possible role in caspase-3 mediated apoptosis. *Heliyon* 8:e12278. 10.1016/j.heliyon.2022.e12278 36590507 PMC9801087

[B96] Trapp J. Meier R. Hongwiset D. Kassack M. Sippl W. Jung M. (2007). Structure-activity studies on suramin analogues as inhibitors of NAD+-dependent histone deacetylases (sirtuins). *ChemMedChem* 2 1419–1431. 10.1002/cmdc.200700003 17628866

[B97] Tsvetkov P. Coy S. Petrova B. Dreishpoon M. Verma A. Abdusamad M. (2022). Copper induces cell death by targeting lipoylated TCA cycle proteins. *Science* 375 1254–1261. 35298263 10.1126/science.abf0529 PMC9273333

[B98] Turc G. Bhogal P. Fischer U. Khatri P. Lobotesis K. Mazighi M. (2019). European Stroke Organisation (ESO) – European Society for Minimally Invasive Neurological Therapy (ESMINT) guidelines on mechanical thrombectomy in acute ischaemic strokeendorsed by stroke alliance for Europe (SAFE). *Eur. Stroke J.* 4 6–12. 10.1177/2396987319832140 31165090 PMC6533858

[B99] Wang F. Shang Y. Zhang R. Gao X. Zeng Q. A. (2019). SIRT1 agonist reduces cognitive decline in type 2 diabetic rats through antioxidative and anti-inflammatory mechanisms. *Mol. Med. Rep.* 19 1040–1048. 10.3892/mmr.2018.9699 30483738 PMC6323206

[B100] Wang P. Guan Y. Du H. Zhai Q. Su D. Miao C. (2012). Induction of autophagy contributes to the neuroprotection of nicotinamide phosphoribosyltransferase in cerebral ischemia. *Autophagy* 8 77–87. 10.4161/auto.8.1.18274 22113203

[B101] Wang P. Lu Y. Han D. Wang P. Ren L. Bi J. (2019). Neuroprotection by nicotinamide mononucleotide adenylyltransferase 1 with involvement of autophagy in an aged rat model of transient cerebral ischemia and reperfusion. *Brain Res.* 1723:146391. 10.1016/j.brainres.2019.146391 31421130

[B102] Xia D. Yuan J. Jiang X. Qi M. Lai N. Wu L. (2021). viaSIRT1 promotes M2 microglia polarization reducing ROS-mediated NLRP3 inflammasome signaling after subarachnoid hemorrhage. *Front. Immunol.* 12:770744. 10.3389/fimmu.2021.770744 34899720 PMC8653696

[B103] Xian W. Li T. Li L. Hu L. Cao J. (2019). Maresin 1 attenuates the inflammatory response and mitochondrial damage in mice with cerebral ischemia/reperfusion in a SIRT1-dependent manner. *Brain Res.* 1711 83–90. 10.1016/j.brainres.2019.01.013 30639123

[B104] Xie W. Zhu T. Zhou P. Xu H. Meng X. Ding T. (2020). Notoginseng leaf triterpenes ameliorates OGD/R-induced neuronal injury via SIRT1/2/3-Foxo3a-MnSOD/PGC-1α signaling pathways mediated by the NAMPT-NAD pathway. *Oxid. Med. Cell. Longev.* 2020:7308386. 10.1155/2020/7308386 PMC7603631 33149812

[B105] Xie X. Cao Y. Dai L. Zhou D. (2023). Bone marrow mesenchymal stem cell-derived exosomal lncRNA KLF3-AS1 stabilizes Sirt1 protein to improve cerebral ischemia/reperfusion injury via miR-206/USP22 axis. *Mol. Med.* 29:3. 10.1186/s10020-022-00595-1 36627572 PMC9830826

[B106] Xing Y. Zhang M. Wang M. Feng Y. Dong F. Zhang F. (2021). The anti-apoptosis effect of single electroacupuncture treatment via suppressing neuronal autophagy in the acute stage of ischemic stroke without infarct alleviation. *Front. Cell. Neurosci.* 15:633280. 10.3389/fncel.2021.633280 33603645 PMC7884854

[B107] Xu Q. Zhou D. Yu D. (2023). Bone marrow mesenchymal stem cells-derived exosomal long non-coding RNA KLF3 antisense RNA 1 enhances autophagy to protect against cerebral ischemia/reperfusion injury via ETS variant transcription factor 4/silent information regulator 1 axis. *Neuroscience* 521 44–57. 10.1016/j.neuroscience.2023.02.021 37080449

[B108] Xu S. Lv H. Li W. Hong H. Peng Y. Zhu B. (2020). Electroacupuncture alleviates cerebral ischemia/reperfusion injury in rats by histone H4 lysine 16 acetylation-mediated autophagy. *Front. Psychiatry* 11:576539. 10.3389/fpsyt.2020.576539 33391046 PMC7775364

[B109] Yan P. Li Z. Xiong J. Geng Z. Wei W. Zhang Y. (2021). LARP7 ameliorates cellular senescence and aging by allosterically enhancing SIRT1 deacetylase activity. *Cell Rep.* 37:110038. 10.1016/j.celrep.2021.110038 34818543

[B110] Yan W. Sun W. Fan J. Wang H. Han S. Li J. (2020). Sirt1-ROS-TRAF6 signaling-induced pyroptosis contributes to early injury in ischemic mice. *Neurosci. Bull.* 36 845–859. 10.1007/s12264-020-00489-4 32253651 PMC7410906

[B111] Yan X. Yu A. Zheng H. Wang S. He Y. Wang L. (2019). OCalycosin-7—-glucoside attenuates OGD/R-induced damage by preventing oxidative stress and neuronal apoptosis via the SIRT1/FOXO1/PGC-1 pathway in HT22 cells. *Neural Plast.* 2019:8798069. 10.1155/2019/8798069 31885537 PMC6915014

[B112] Yang R. Shen Y. Chen M. Zhao J. Chen S. Zhang W. (2022). Quercetin attenuates ischemia reperfusion injury by protecting the blood-brain barrier through Sirt1 in MCAO rats. *J. Asian Natl. Products Res.* 24 278–289. 10.1080/10286020.2021.1949302 34292112

[B113] Yang W. Tao K. Zhang P. Chen X. Sun X. Li R. (2022). Maresin 1 protects against lipopolysaccharide/d-galactosamine-induced acute liver injury by inhibiting macrophage pyroptosis and inflammatory response. *Biochem. Pharmacol.* 195:114863. 10.1016/j.bcp.2021.114863 34861244

[B114] Yang X. Si P. Qin H. Yin L. Yan L. Zhang C. (2017). The neuroprotective effects of SIRT1 on NMDA-induced excitotoxicity. *Oxid. Med. Cell. Longev.* 2017:2823454. 10.1155/2017/2823454 PMC5610841 29081884

[B115] Yang X. Sun X. Wu J. Ma J. Si P. Yin L. (2020). Regulation of the SIRT1 signaling pathway in NMDA-induced Excitotoxicity. *Toxicol. Lett.* 322 66–76. 31945382 10.1016/j.toxlet.2020.01.009

[B116] Yang Z. Huang C. Wen X. Liu W. Huang X. Li Y. (2022). Circular RNA circ-FoxO3 attenuates blood-brain barrier damage by inducing autophagy during ischemia/reperfusion. *Mol. Therapy* 30 1275–1287. 10.1016/j.ymthe.2021.11.004 34763084 PMC8899525

[B117] Ye J. Zhang F. Li B. Liu Q. Zeng G. (2022). Knockdown of ATF3 suppresses the progression of ischemic stroke through inhibiting ferroptosis. *Front. Mol. Neurosci.* 15:1079338. 10.3389/fnmol.2022.1079338 36743288 PMC9890179

[B118] Yeung F. Hoberg J. Ramsey C. Keller M. Jones D. Frye R. (2004). Modulation of NF-kappaB-dependent transcription and cell survival by the SIRT1 deacetylase. *EMBO J.* 23 2369–2380. 10.1038/sj.emboj.7600244 15152190 PMC423286

[B119] Yu H. Kim Y. Cho M. (2020). Cytoplasm-localized SIRT1 downregulation attenuates apoptosis and cell cycle arrest in cisplatin-resistant lung cancer A549 cells. *J. Cancer* 11 4495–4509. 10.7150/jca.44383 32489467 PMC7255359

[B120] Yu H. Zhang F. Yan P. Zhang S. Lou Y. Geng Z. (2021). LARP7 protects against heart failure by enhancing mitochondrial biogenesis. *Circulation* 143 2007–2022. 10.1161/CIRCULATIONAHA.120.050812 33663221

[B121] Yu P. Wang L. Tang F. Guo S. Liao H. Fan C. (2021). Resveratrol-mediated neurorestoration after cerebral ischemic injury – Sonic Hedgehog signaling pathway. *Life Sci.* 280:119715. 10.1016/j.lfs.2021.119715 34116113

[B122] Yu P. Wang L. Tang F. Zeng L. Zhou L. Song X. (2017). Resveratrol pretreatment decreases ischemic injury and improves neurological function via sonic hedgehog signaling after stroke in rats. *Mol. Neurobiol.* 54 212–226. 10.1007/s12035-015-9639-7 26738852

[B123] Yue L. Zhao L. Liu H. Li X. Wang B. Guo H. (2016). Adiponectin protects against glutamate-induced excitotoxicity via activating SIRT1-dependent PGC-1α expression in HT22 hippocampal neurons. *Oxid. Med. Cell. Longev.* 2016:2957354. 10.1155/2016/2957354 PMC5155107 28042384

[B124] Zakhary S. Ayubcha D. Dileo J. Jose R. Leheste J. Horowitz J. (2010). Distribution analysis of deacetylase SIRT1 in rodent and human nervous systems. *Anatomical Rec.* 293 1024–1032. 10.1002/ar.21116 20225204 PMC3071026

[B125] Zhang M. Lu P. Terada T. Sui M. Furuta H. Iida K. (2021). Quercetin 3,5,7,3’,4’-pentamethyl ether from Kaempferia parviflora directly and effectively activates human SIRT1. *Commun. Biol.* 4:209. 10.1038/s42003-021-01705-1 33608631 PMC7896056

[B126] Zhang X. Zhang F. Yao F. Wang P. Xiong Q. Neng P. (2022). Bergenin has neuroprotective effects in mice with ischemic stroke through antioxidative stress and anti-inflammation via regulating Sirt1/FOXO3a/NF-κB signaling. *Neuroreport* 33 549–560. 36049159 10.1097/WNR.0000000000001789

[B127] Zhang Y. Yan Y. Cao Y. Yang Y. Zhao Q. Jing R. (2017). Potential therapeutic and protective effect of curcumin against stroke in the male albino stroke-induced model rats. *Life Sci.* 183 45–49. 10.1016/j.lfs.2017.06.023 28663065

[B128] Zhao K. Zhang Y. Xu X. Liu L. Huang L. Luo R. (2019). Acetylation is required for NLRP3 self-aggregation and full activation of the inflammasome. *bioRxiv* [preprint]. 10.1101/2019.12.31.891556

[B129] Zhao Y. Shi X. Wang J. Mang J. Xu Z. (2021). Betulinic acid ameliorates cerebral injury in middle cerebral artery occlusion rats through regulating autophagy. *ACS Chem. Neurosci.* 12 2829–2837. 10.1021/acschemneuro.1c00198 34296845

[B130] Zheng X. Shan C. Xu Q. Wang Y. Shi Y. Wang Y. (2018). Buyang huanwu decoction targets SIRT1/VEGF pathway to promote angiogenesis after cerebral ischemia/reperfusion injury. *Front. Neurosci.* 12:911. 10.3892/mmr.2021.12431 30564092 PMC6288378

[B131] Zheng Y. Li L. Chen B. Fang Y. Lin W. Zhang T. (2022). Chlorogenic acid exerts neuroprotective effect against hypoxia-ischemia brain injury in neonatal rats by activating Sirt1 to regulate the Nrf2-NF-κB signaling pathway. *Cell Commun. Signal.* 20:84. 10.1186/s12964-022-00860-0 PMC9185968 35689269

[B132] Zhou D. Chen L. Wang Y. Gan L. Yuan M. Zhang L. (2023). RNA binding protein RPS3 mediates microglial polarization by activating NLRP3 inflammasome via SIRT1 in ischemic stroke. *J. Stroke Cerebrovasc. Dis.* 32:107132. 10.1016/j.jstrokecerebrovasdis.2023.107132 37087770

[B133] Zhou L. Zhu H. Bai X. Huang J. Chen Y. Wen J. (2022). Potential mechanisms and therapeutic targets of mesenchymal stem cell transplantation for ischemic stroke. *Stem Cell Res. Therapy* 13:195. 10.1186/s13287-022-02876-2 PMC9096773 35551643

[B134] Zhou S. Qiao B. Chu X. Kong Q. (2018). Oxymatrine attenuates cognitive deficits through SIRT1-mediated autophagy in ischemic stroke. *J. Neuroimmunol.* 323 136–142. 10.1016/j.jneuroim.2018.06.018 30196826

[B135] Zhou Y. Li G. (2020). Kaempferol protects cell damage in in vitro ischemia reperfusion model in rat neuronal PC12 cells. *BioMed Res. Int.* 2020:2461079. 10.1155/2020/2461079 32382538 PMC7196139

[B136] Zhu H. Huang J. Chen Y. Li X. Wen J. Tian M. (2022). Resveratrol pretreatment protects neurons from oxygen-glucose deprivation/reoxygenation and ischemic injury through inhibiting ferroptosis. *Biosci. Biotechnol. Biochem.* 86 704–716. 10.1093/bbb/zbac048 35357412

[B137] Zhu T. Xie W. Wang L. Jin X. Meng X. Sun G. (2021). Notoginsenoside R1 activates the NAMPT-NAD-SIRT1 cascade to promote postischemic angiogenesis by modulating Notch signaling. *Biomed. Pharmacother.* 140:111693. 10.1016/j.biopha.2021.111693 34029951

